# A Longitudinal Cohort Study Investigating Inadequate Preparation and Death and Dying in Nursing Students: Implications for the Aftermath of the COVID-19 Pandemic

**DOI:** 10.3389/fpsyg.2020.02206

**Published:** 2020-08-25

**Authors:** John Galvin, Gareth Richards, Andrew Paul Smith

**Affiliations:** ^1^Department of Psychology, Birmingham City University, Birmingham, United Kingdom; ^2^School of Psychology, Newcastle University, Newcastle upon Tyne, United Kingdom; ^3^School of Psychology, Cardiff University, Cardiff, United Kingdom

**Keywords:** nursing students, mental health, death and dying, COVID-19, longitudinal

## Abstract

**Aims and Objectives:**

To investigate how changes in the levels of preparedness and experiences of death and dying influence nursing students’ mental health.

**Background:**

The COVID-19 pandemic is likely to cause significant trauma in the nursing population. The lack of preparation, in combination with a substantial loss of life, may have implications for the longer-term mental health of the nursing workforce. Nursing students have, in many cases, been an important part of the emergency response.

**Design:**

A longitudinal cohort study was conducted in the academic year 2014/15 with data collected at two time points. There was a 7-month time period between data collection.

**Methods:**

Participants completed paper-based questionnaires measuring demographics, academic stressors, clinical stressors, and mental health. 358 nursing students at time point one and 347 at time point two (97% retention) completed the survey.

**Results:**

Inadequate preparation (OR: 1.783) and the inadequate preparation x death and dying interaction term (OR: 4.115) significantly increased risk of mental health problems over time. Increased death and dying alone did not increase mental health risk.

**Conclusion:**

The results of this study suggest that it is not the increase in death and dying *per se* that causes mental health difficulties, but that it is instead the experience of high levels of death and dying in combination with inadequate preparation. The data are considered within the context of the COVID-19 pandemic, with both inadequate preparation and the scale of death and dying being two significant stressors during the emergency period.

## Introduction

The well-being of healthcare workers is at the forefront of debates during the COVID-19 public health emergency (e.g., Greenberg et al., 2020; Tanne et al., 2020). High volumes of patients are being admitted to hospitals across the world and it is difficult to keep count of, or be able to predict, the total number of deaths on a national, regional or global level. As of 15th July 2020, the Coronavirus Resource Center at John Hopkins University reported 13,357,992 confirmed cases and 579,508 deaths worldwide. Early indications suggest that psychiatric disorder is likely following the pandemic (Moccia et al., 2020; Sani et al., 2020). For example, a cross-sectional study in China (Lai et al., 2020) found that healthcare workers are reporting high rates of insomnia, anxiety and depressive symptoms during the emergency period. Outcomes were poorer in nurses, females, frontline health care workers, and those working in the city of Wuhan, China. Nurses are working at the forefront of this pandemic, working in very challenging environments and with shortages of personal protective equipment (PPE), medical supplies, and essential machinery such as ventilators (Ranney et al., 2020). The increased volume of deadly cases across the world is likely to cause significant trauma in the nursing population, with implications for the longer-term mental health of the workforce.

The nursing workforce has required rapid expansion in a short period of time to help with the demand. In the United Kingdom, recently retired nurses and undergraduate nursing students are being recruited as part of the emergency response. The United Kingdom regulator of nurses and midwives, the Nursing and Midwifery Council (2020), has permitted third-year undergraduate nursing students in their final 6 months of training to be considered for a temporary NMC registration and to be deployed in an enhanced role in the National Health Service (NHS). Students have therefore prematurely become part of the workforce.

This paper presents previously unpublished data collected during the first author’s doctoral research (Galvin, 2016) in the academic year 2014/15. In this study, longitudinal data on nursing students’ experiences of death and dying, their feelings of preparedness, and subsequent mental health was collected. These data are extremely relevant in the context of COVID-19, because nursing students have now prematurely been pulled away from their studies to support with the crisis and may lack adequate preparation to deal with such an unprecedented number of deaths. In the context of the pandemic, we therefore returned to this data with some specific hypotheses in mind.

## Background

The term stress refers to any demand, event or situation that disturbs the adaptive state and threatens to exceed the individual’s resources and skills; it is a dynamic, transactional, relationship between the individual and their environment (Lazarus, 1999). If the individual’s adaptive state is altered by an event, this may provoke a coping response. Inappropriate coping responses have repercussions for mental health (Karaca et al., 2019).

The emotional issues resulting from the death and suffering of patients are important stressors for nurses (Lambert et al., 2004; Burnard et al., 2008). Wang (2019) conducted a systematic review of qualitative studies investigating nursing students’ experiences when caring for dying patients. The review found that students can experience a wide range of negative emotions including helplessness, feelings of incompetence, guilt, self-doubt, fear, and anxiety. To cope with such emotions, students report both positive and negative coping strategies. Negative coping strategies included suppressing any felt sadness, emotionally distancing themselves from the dying patient, and avoidance coping (Charalambous and Kaite, 2013; Österlind et al., 2016). Positive strategies included talking with other students who had similar experiences, talking with family, and allowing themselves to cry (Cooper and Barnett, 2005; Garrino et al., 2017).

Although there is currently a lack of available data on the sources and levels of stress amongst nurses during the COVID-19 pandemic, it is likely that nurses and nursing students are experiencing significant levels of emotional distress exacerbated by several salient factors. These include, but are certainly not limited to, staff and resource shortages, longer working hours, clinical uncertainty associated with a lack of clinical guidelines, care rationing, unpredictability surrounding the timescale of the pandemic, risk of personal illness or death, and the scale of disease and death being encountered (Jackson et al., 2020; Smith et al., 2020; Usher et al., 2020). In the wake of the pandemic, COVID-related stressors have had a rapid onset, with little time for preparation.

Jackson et al. (2020) described how nurses were bracing themselves for a “tsunami of death” (p. 2), and there is clear risk that the personal and professional development of nursing students may be tossed aside in this storm. It remains unknown how this pandemic will affect the students working during the emergency period, their relationship with death, and how they deal with dying patients in the future. It is unlikely that the situation will allow them to prepare adequately and reflect or establish meaningful relationships with patients who are dying, which are important aspects of nurses’ personal and professional development (Henoch et al., 2017) because they help eliminate students’ anxieties about death (Adesina et al., 2014).

### Aims and Hypothesis

This paper describes a longitudinal cohort study investigating the relationship between feelings of preparedness, death and dying, and risk of mental health problems in nursing students. It was predicted that (i) feelings of inadequate preparation between the two time points will be significantly associated with an increased risk of mental health problems; (ii) increased death and dying between the two time points will be significantly associated with increased risk of mental health problems; and (iii) a significant interaction will be found between levels of preparation and death and dying; more specifically, we predict that a combination of inadequate preparation and increased experience of death and dying will predict increased occurrence of mental health difficulties.

## Methods

The longitudinal cohort study was based at a Higher Education Institution in Wales, United Kingdom and was granted ethical approval by the School of Psychology Research Ethics Committee (REC). The Healthcare REC at the same institution honored the Psychology REC and granted permission to approach their students to take part in the study.

The work described in this paper has been carried out in accordance with The Code of Ethics of the World Medical Association (Declaration of Helsinki). The research involved human participants, all of whom provided informed consent before taking part in the study. The research methods were compliant with the Strengthening the Reporting of Observational Studies in Epidemiology (STROBE) checklist (see appendix 1).

### Data

Data were collected from nursing students enrolled on a 3-year undergraduate Bachelor of Nursing degree during the 2014/15 academic year. At time point 1 (T1), participants completed a survey in the middle of their first clinical placement of the academic year, and at time point 2 (T2), they completed the same survey at the end of the academic year. There was a 7-month time period between data collection at T1 and T2.

This was a paper-based survey and students were approached in lecture theaters at the host institution. T1 data were collected on a day in which students returned to the university half-way through their clinical placements, whereas T2 data were collected during the final academic lecture of the year. The survey was distributed at the beginning of the lectures, with participants completing and returning the survey during the break.

### Measurements

Participants reported basic demographic information (sex, ethnicity, age, relationship status and year of training) before completing a series of questionnaire measures. The Student Short-Version of the Wellbeing Process Questionnaire (SS-WPQ; Williams G. M. et al., 2017) and an adapted version of the Nurse Stress Scale (NSS, Gray-Toft and Anderson, 1981) designed for students (i.e., the student-nurse stress scale; S-NSS; Galvin, 2016) were administered at both time points. The scales have been developed in previous work by our research group and demonstrate good psychometric properties (e.g., see Williams, 2014; Galvin, 2016; Williams G. et al., 2017; Williams G. M. et al., 2017, Williams and Smith, 2018; Smith and Firman, 2020).

The SS-WPQ is comprised of seven factors related to student stressors (challenges to your development, time pressures, academic dissatisfaction, romantic problems, societal annoyances, social mistreatment, and friendship problems). Responses are recorded on a scale of 1–10, where 1 indicated “not at all a part of my life” and 10 indicates “very much a part of my life”. For the outcome measure, the mental health scale of the SS-WPQ was used, which is a sum score of three factors: anxiety, depression, and happiness (reverse scored).

The S-NSS comprises stressors related to clinical placements, and the following factors were included: being inadequately prepared, lack of support, conflict with other nurses, heavy workload, death and dying, conflict with other staff, discrimination, and hassles from patients/relatives. Responses were again recorded on a scale of 1–10, and students were asked to indicate the frequency by which they had experienced each source of stress (1 indicating “never” and 10 indicating “very frequently”).

### Data Analysis

Data were analyzed using IBM SPSS version 25. After normality checks and initial descriptive analyses of the demographic variables, change scores were computed for all factors. Multivariate logistic regression analysis (enter method) was used to assess mental health risk. The model included each independent variable as well as all demographic variables as covariates. Effects were considered significant if they met a threshold of *p* < 0.05. A manual backward-step approach was taken, and all factors from the final model which met a threshold of *p* > 0.10 were excluded, to ensure the inclusion of marginally non-significant effects.

## Results

At the beginning of the academic year, 573 nursing students were enrolled on the nursing course. At T1, the sample size was 358 (response rate = 62.5%). At the end of the academic year, 557 students were enrolled on the course, and at T2, the sample size was 347 (response rate = 62.3%). It is likely that the actual response rates will have been higher than reported here, as these are somewhat conservative estimates. For example, information on the number of students that enrolled at the beginning of the academic year but did not actually attend the course was not available. The study also did not have data on the number of students who were taking a temporary withdrawal from their studies. Eleven students who had completed the survey at T1 did not complete the survey at T2, resulting in a 97% retention rate. Descriptive data for the sample is reported in Table 1. Most participants were female, married or in a relationship, and of White ethnicity. Seventeen per cent of the sample reported never having experienced death and dying whilst on placement at T1, and this figure reduced to 13% at T2.

**TABLE 1:** Characteristics of the sample at T1 and T2.

Characteristic	T1	T2
Sex	314 (87%) Female	307 (87%) Female
	44 (13%) Male	40 (13%) Male
Age *M* (SD), Range	*25.21* (6.48), 18–48 years	*25.65* (6.47), 18–49 years
Ethnicity	335 (94%) White	325 (94%) White
Relationship status	185 (53%) in a relationship or married	185 (52%) in a relationship or married
Year of training	Year 1 = 88 (26%)	Year 1 = 85 (26%)
	Year 2 = 147 (41%)	Year 2 = 143 (42%)
	Year 3 = 123 (33%)	Year 3 = 119 (32%)

Paired samples *t*-tests comparing T1 and T2 on inadequate preparation, death and dying, and mental health scores were all non-significant (*p* > 0.05). Manual backward-step logistic regression was then conducted (Table 2) with mental health risk between T1 and T2 as a dichotomous outcome (1 = increased risk, 0 = no increased risk). Statistically significant direct effects were observed for the following factors: academic dissatisfaction (OR: 2.675), friendship problems (OR: 2.165), and inadequate preparation (OR: 1.783). A significant interaction was found between feeling inadequately prepared for placements and experience of death and dying on placements (OR: 4.115).

**TABLE 2:** Results of the manual backward-step logistic regression analysis with mental health as outcome (>risk vs ≤risk).

Outcome: mental health	OR	95% CI	*P*	Wald
Challenges to development	1.591	0.990–2.556	0.055	3.686
Time pressures	1.587	0.989–2.547	0.055	2.547
Academic dissatisfaction	2.675	1.618–4.421	< 0.001	4.421
Friendship problems	2.165	1.357–3.453	0.001	10.501
Lack of support	1.809	1.120–2.923	0.015	5.868
Inadequately prepared	1.783	1.098–2.895	0.021	5.458
Death and dying*	0.911	0.575–1.442	0.690	0.159
Interaction term: Inadequately prepared X death and dying*	4.115	1.575–10.748	0.004	8.340

*Non-significant factors were removed from the model in the following order: Ethnicity, relationship status, hassles from patients/relatives, conflict with other staff, sex, romantic problems, societal annoyances, social mistreatment, year of training, age, conflict with other nurses, heavy workload, discrimination. ^∗^Death and dying refers to the reported number of experiences of death and dying between the two time points. OR, odds ratio, CI, confidence interval.*

**FIGURE 1:**
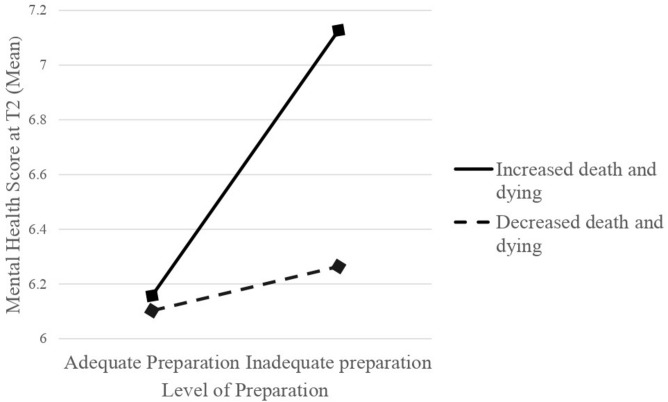
Interaction between the reported feelings of preparation and experience of death and dying on the outcome of mental health score (Mean) at T2.

A dummy variable was then created, with participants being categorized into four groups depending on levels of preparation and experience of death and dying between T1 and T2 (see Table 3). A one-way ANOVA with Bonferroni *post hoc* testing was carried out with T2 mental health score as outcome to determine whether there were differences between the groups (results also presented in Table 3). Participants who reported increased inadequate preparation and increased death and dying between T1 and T2 were at an increased risk of mental health problems at T2 (Figure 1).

**TABLE 3:** Group analysis, including *n* (%), means, standard deviations (for mental health; scale range 1–10), ANOVA and Bonferroni *post hoc*.

Group	*N* (%)	Mean	SD	Statistic	*Post hoc*
Adequately prepared + >death and dying	89 (25.6)	6.123	2.120	F = 3.365**	3 > 1*
					3 > 2*
Adequately prepared + ≤death and dying	127 (36.6)	6.102	2.111		
Inadequately prepared + >death and dying	63 (18.2)	7.127	2.518		
Inadequately prepared + ≤death and dying	68 (19.6)	6.264	2.354		

*≤ = fewer or the same number of experiences of death and dying at T2 compared to T1, > = an increase in the number of experiences of death and dying at T2 compared to T1. ^∗^p ≤ 0.05; ^∗∗^p ≤ 0.01; ^∗∗∗^p ≤ 0.001.*

## Discussion

Clinical placements will expose nursing students to a variety of potential stressors that may negatively impact their mental health (Alzayyat and Al-Gamal, 2014; Galvin et al., 2015). Although a large body of literature on the sources and levels of stress across diverse groups of nursing students exists (e.g., Labrague et al., 2017, 2018; Bhurtun et al., 2019), there remains a lot to learn about the consequences of the COVID-19 pandemic for nurses and students working during the emergency period. An equally large body of literature will undoubtedly emerge regarding its impact. However, because the development of this research in the aftermath of the pandemic is likely to be slow and challenging (e.g., inability to conduct face-to-face research and other data collection restrictions), it is necessary to rely on existing data to ensure that early intervention is appropriately directed and most effective.

The findings of this study supported two of the three hypotheses, with inadequate preparation (Hypothesis 1; OR: 1.783) and the inadequate preparation x death and dying interaction term (Hypothesis 3; OR: 4.115) both being statistically significant predictors of increased risk of mental health problems. Increased death and dying alone did not increase mental health risk (Hypothesis 2: OR: 0.911). Additional risk factors in our final model, and potentially of relevance to COVID-19 included: time pressures (OR: 1.587), challenges to development (OR: 1.591), friendship problems (OR: 2.165), and a lack of support (OR: 1.809).

Although the current study did not find support for hypothesis 2, the data on death and dying in this research are not comparable to the scale of death and dying during the COVID-19 pandemic, and it is likely that the present study reflects an under-estimate of the true effect. Our data do suggest, however, that it is not the increase in death and dying *per se* that causes mental health difficulties, but that it is instead the experience of high levels of death and dying in combination with inadequate preparation. The COVID-19 situation has resulted in the whole workforce falling within this latter situation, with both inadequate preparation and the scale of death being two significant stressors during the emergency period (Jackson et al., 2020; Smith et al., 2020; Usher et al., 2020).

Significant trauma and longer-term implications from this pandemic are expected to impact the nursing workforce as well as the profession more generally. The scale of the trauma is hard to judge at this point, but our findings are an indication of the potential scale of the problems to come. Plans need to be developed, with adequate protections for both staff and student wellbeing through early intervention and increased resources. For the year following the pandemic, our recommendations for supporting healthcare staff and students include: access to specialist services for post-traumatic stress; counseling and mental health support through a variety of media (e.g., telephone, apps, online, face-to-face); peer and network support programs specifically designed for COVID-19; access to peer-to-peer supervision and adjusted workload to allow attendance; introduction of an emotional element to shift handovers; repurposing offices into rest spaces; additional “recovery” holidays and extended sick pay (in the case of students, greater flexibility for assessments); and raising awareness of how to access support (e.g., key contacts). The scope, level and necessity of support will of course be dependent on location (country and region/county/city), suggesting that local services should consult with their health and safety representatives, public health and occupational health colleagues to develop a local plan to support the workforce.

Although these findings are interesting within the context of the COVID-19 pandemic, it is important to note that this is a preliminary study and based on data collected five years ago. The generalizability of the findings can therefore be questioned. However, in the context of limited information regarding the impact of the pandemic, and the urgent need for workforce planning, we argue its relevance. Future research focused on the impact of the COVID-19 emergency on the mental health of nursing students working during the emergency period should explore these relationships further.

Further limitations of the study include the timing of the survey at T1, as it was in the middle of the first clinical placement and therefore first year students had minimal clinical experience (6 weeks). It may have been preferable to wait until the end of the first clinical placement to allow greater clinical exposure. This limitation is likely to have affected some of the variables of interest for the year one subsample. For example, at the first time point 17% of the overall sample had not experienced death and dying on placement, and, at the second time point this figure fell to 13%. We controlled for this issue as well as we could with the inclusion of year group as a covariate, and, although these figures are higher than those observed by some studies (e.g., 6% in Henoch et al., 2017), they are lower than those reported by others (e.g., 22% in Mutto et al., 2010). Additionally, having all the students in the university at the same time was convenient, and approaching participants at a later date would have resulted in a reduced sample size and shorter duration between the two data collection points.

## Conclusion

The present research found no evidence of an association between increased experiences of death and dying and mental health outcomes. However, a combination of inadequate preparation and increased death and dying was associated with increased mental health problems across the 7-month time period. The variables included in this study are of particular interest during these unprecedented times, but further work in this area is necessary.

## Relevance to Clinical Practice

COVID-19 has disrupted nursing students’ education, and for many, has required an overnight shift from the requirements and expectations of a learner, to the requirements and expectations of a worker. The findings of this study serve as an initial preliminary indication of the possible longer-term mental health impact of COVID-19 for nursing students and highlights the need for strategic planning. It was impossible to be fully prepared for this pandemic, but we can be prepared for its consequences. Intervention and resource planning should therefore be based on location, ensuring immediate support is provided to workers in the areas with the highest number of deadly cases.

## Data Availability Statement

The raw data supporting the conclusions of this article will be made available by the authors, without undue reservation.

## Ethics Statement

The studies involving human participants were reviewed and approved by the Research Ethics Committee of the School of Psychology, Cardiff University. The patients/participants provided their written informed consent to participate in this study.

## Author Contributions

JG was involved in the conception and design of the study, carried out the statistical analyses, interpreted the data, and drafted and revised the manuscript. AS supervised the project and contributed knowledge and experience to the conception, design, and analysis stages of the research. GR supported with data collection and revising of the article for important intellectual content. All authors read and approved the final manuscript.

## Conflict of Interest

The authors declare that the research was conducted in the absence of any commercial or financial relationships that could be construed as a potential conflict of interest.
